# Model-Based Assessment of Dosing Strategies in Children for Monoclonal Antibodies Exhibiting Target-Mediated Drug Disposition

**DOI:** 10.1038/psp.2014.38

**Published:** 2014-10-01

**Authors:** S Zheng, P Gaitonde, M A Andrew, M A Gibbs, L J Lesko, S Schmidt

**Affiliations:** 1 Center for Pharmacometrics and Systems Pharmacology, University of Florida, Orlando, Florida, USA; 2 Department of Pharmacokinetics and Drug Metabolism, Amgen, Seattle, Washington, USA; 3 Department of Pharmacokinetics and Drug Metabolism, Amgen, Thousand Oaks, California, USA

## Abstract

Body weight/body surface area–based and/or tiered fixed dosing strategies are widely utilized for monoclonal antibodies with linear clearance to scale adult clinical doses to children. However, there is limited knowledge on whether or not body weight–based dosing strategies also yield comparable dose-concentration-response relationships in adults and children for monoclonal antibodies that exhibit target-mediated drug disposition. Our findings indicate that it is important to interpret pharmacokinetics information in a pharmacokinetics/pharmacodynamics context as similar systemic drug exposure in adults and children may not be reflective of the corresponding target occupancy. They further indicate that BW-based dosing is superior to fixed dosing for the same target concentration, whereas the opposite holds true for the same target amount in adults and children. Michaelis-Menten approximations yielded similar profiles compared to the full target-mediated drug disposition model for all simulation scenarios and may be used to guide the selection of appropriate dosing regimens in children.

In recent years, pediatric drug development has been encouraged by regulatory authorities in light of the insufficient number of drugs, doses and formulations available for application in children and high off-label use of adult medicines.^1^ The US Food and Drug Administration Safety and Innovation Act (FDASIA) 2012 requires sponsors to submit a pediatric drug development plan to the regulatory agency at the end of phase II studies^2^ while a Pediatric Investigation Plan (PIP) is required earlier in Europe at the end of Phase I studies.^3^ Although the establishment of pediatric development plans is in many cases challenging due to the lack of data and an incomplete understanding of the drug's pharmacokinetics (PK) and pharmacodynamics (PD) in children, these regulatory initiatives prompted the early use of predictive models to support pediatric drug development programs.^4,5,6,7^ These models frequently employ simple allometric functions to scale a PK parameter (*Y*), such as clearance or volume of distribution, from adults to children using a body-weight (BW)–based power function (*Y* = *a·*BW^*b*^) with a coefficient (*a*) and an exponent (*b*).^8,9^ Although this scaling approach has found wide application for small molecules,^9^ it has its limitations for capturing highly non-linear processes, such as target-mediated drug disposition (TMDD) of monoclonal antibodies (mAbs)^10,11^ or maturational changes in mAb disposition from birth.^12^

Therapeutic mAbs are cleared via multiple routes, which can be divided into mAb-specific and mAb non-specific elimination routes. Non-specific mAb elimination is primarily mediated by intracellular catabolism following fluid phase and receptor-mediated endocytosis,^8^ which is typically non-saturable at therapeutic doses (i.e., linear PK). Interactions between the mAb and its specific target, on the other hand, can lead to saturable and thus non-linear PK. Low mAb concentration relative to the concentration of target result in rapid elimination.^13^ Once mAb concentrations increase, more targets are occupied, which results in a non-linear decrease in clearance. At very high mAb concentrations, when targets are completely saturated, clearance can be considered linear again.^14^ As such, BW-based allometric scaling may be sufficient to scale adult doses to children for mAbs with linear kinetics except for low weight children,^15,16^ but may less accurately predict pediatric dosing regimens for mAbs that show non-linear kinetics, i.e., that employ TMDD. Literature adult data suggests that PK parameters for many mAbs change in a less than BW-proportional manner as respective allometric exponents for clearance and volume of distribution were estimated to range from 0.3 to 0.7 (refs. 17,18) There are only a few examples, where strong BW effects were observed as indicated by exponents greater than 0.75.^17^ It should be noted that allometric exponents determined for within species scaling are typically smaller than those observed for between species scaling, which is likely the result of a narrower BW range in adults for a given species (approximately two- to threefold).^17^ Since the respective BW range in children is wider than that in adults, further research is necessary to determine appropriate allometric exponents in children.^17^ For example, clearance exponents were estimated to be 0.823 (ref. ^19^) for canakinumab in systemic juvenile idiopathic arthritis (SJIA) patients, whereas a value of 0.75 was found appropriate for palivizumab and infliximab when scaling from adults to children.^15,20^ Yet, few studies systematically explored the entire range of allometric exponents to determine an optimal value for CL and V_ss_ across mAbs. There is also limited information on whether or not BW-based dosing strategies allow to reliably predict pediatric doses that result in comparable systemic drug exposure (AUC_0–infinity_) in children and adults for mAbs that exhibit TMDD. The uncertainty around how to optimally scale adult doses to children for mAbs with non-linear kinetics is reflected in the lack of approved pediatric dosing regimens as compared to mAb with linear kinetics (**Table 1**).

The objective of our study was therefore to evaluate the impact of differences in target expression between adults and children on pediatric dosing for mAbs exhibiting non-linear kinetics via simulations.

## Results

### Same target concentration: BW-based dosing

**Figure 1:**
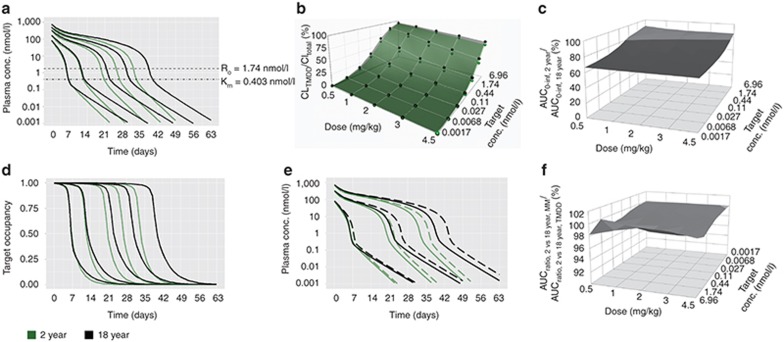
Same target concentration, body weight (BW)-based intravenous (i.v.) dosing. (**a**) Simulated plasma PK profiles for 2-year (green lines) vs. 18-year (black lines) age group (reference group) based on the full TMDD model at doses of 0.5, 1, 2, 3, and 4.5 mg/kg (from left to right). (**b**) CL_TMDD_/CL_total_ for 2-year (green dots connected with the green surface) vs. 18-year age group (black dots connected with the gray surface). (**c**) AUC_0–infinity_ ratios for 2-year vs. 18-year age group. (**d**) Simulated target occupancy for 2-year (green lines) vs. 18-year (black lines) age group at doses of 0.5, 1, 2, 3, and 4.5 mg/kg (from left to right). (**e**) Comparison of simulated plasma PK profiles by the full TMDD model (solid line) and its Michaelis-Menten approximations (dashed line) in the 2-year (green lines) and 18-year (black lines) age group at doses of 0.5, 2, and 4.5 mg/kg (from left to right). (**f**) Relative change in AUC ratios between 2-year-olds and 18-years olds when comparing MM approximations and full TMDD model. The target concentration (1.74 nmol/l) was reported in Luu *et al.*^31^ Sensitivity analysis of target concentration *R*_0_ was performed by using a range of hypothetical *R*_0_ values, which were changed in fourfold increments (6.96, 1.74, 0.44, 0.11, 0.027, 0.0068, and 0.0017 nmol/l).

At the same target concentration (1.74 nmol/l), mAb PK was similar between adults and children at all doses (**Figure 1a**). At target concentrations above 0.44 nmol/l, CL_TMDD_ significantly contributed to the mAb's total clearance (CL_TOT_) at all doses and both age groups (**Figure 1b**). At 1.74 nmol/l target baseline concentration (*R*_0_), systemic mAb exposure in children (AUC_0–infinity_) decreased from 86% to 78% of that in adults from 0.5 to 4.5 mg/kg dosing (**Figure 1c**). However, target occupancy did not differ significantly for the lowest two doses and was maintained above 90% in the 2-year-olds for 31 days (84% of the duration in the 18 year olds) at 4.5 mg/kg (**Figure 1d**). Once combined, plasma PK and target occupancy correlated reasonably well with each other. Michaelis-Menten (MM) approximations resulted in drug PK comparable to the full TMDD model for both age groups, particularly at the high concentration range (**Figure 1e**). The estimated AUC ratios between 2- and 18-year-old based on MM approximations were in good agreement with the estimated AUC ratios based on the full TMDD model for all doses across the range of target concentrations (**Figure 1f**). Similar findings were seen for the 6- and 12-year age group (data not shown).

### Same target concentration: fixed dosing

**Figure 2:**
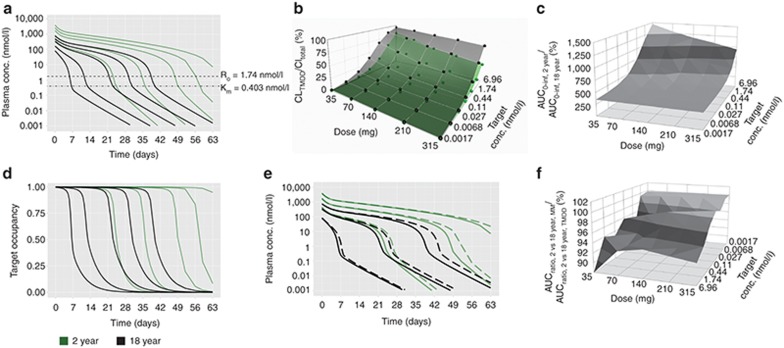
Same target concentration, fixed i.v. dosing. (**a**) Simulated plasma PK profiles for 2-year (green lines) vs. 18-year (black lines) age group (reference group) based on the full TMDD model at doses of 35, 70, 140, 210, and 315 mg (from left to right). (**b**) CL_TMDD_/CL_total_ for 2-year (green dots connected with the green surface) vs. 18-year age group (black dots connected with the gray surface). (**c**) AUC_0–infinity_ ratios for 2-year vs. 18-year age group. (**d**) Simulated target occupancy for 2-year (green lines) vs. 18-year (black lines) age group at doses of 35, 70, 140, 210, and 315 mg (from left to right). (**e**) Comparison of simulated plasma PK profiles by the full TMDD model (solid line) and its Michaelis-Menten approximations (dashed line) in the 2-year (green lines) and 18-year (black lines) age group at doses of 35, 140, and 315 mg (from left to right). (**f**) Relative change in AUC ratios between 2-year-olds and 18-years olds when comparing MM approximations and full TMDD model. The target concentration (1.74 nmol/l) was reported in Luu *et al.*^31^ Sensitivity analysis of target concentration *R*_0_ was performed by using a range of hypothetical *R*_0_ values, which were changed in fourfold increments (6.96, 1.74, 0.44, 0.11, 0.027, 0.0068, and 0.0017 nmol/l).

Fixed dosing for same target concentrations (1.74 nmol/l) between pediatrics and adults resulted in higher systemic mAb concentrations in children (**Figure 2a**). The smaller volume of distribution in younger children resulted in higher *C*_max_ values at all doses. CL_TMDD_ largely accounted for CL_TOT_ in adults compared to pediatrics at the lowest dose (**Figure 2b**) but its contribution decreased in both age-groups with increasing dose. AUC ratio differences were prominent at the lowest dose (35 mg) and highest target concentration (6.96 nmol/l) (**Figure 2c**) due to target saturation in pediatrics. Consequently, duration of target occupancy was significantly prolonged for all doses in pediatrics compared to adults (**Figure 2d**). Similar to BW-based dosing, plasma PK were reflective of target occupancy. PK profiles generated by MM approximations were similar to those obtained from the full TMDD model for both age groups (**Figure 2e**). MM- or Full TMDD-based AUC ratios between 2- and 18-year-old were comparable (88–102%) for all doses across the range of target concentrations (**Figure 2f**).

### Same target amount: BW-based dosing

**Figure 3:**
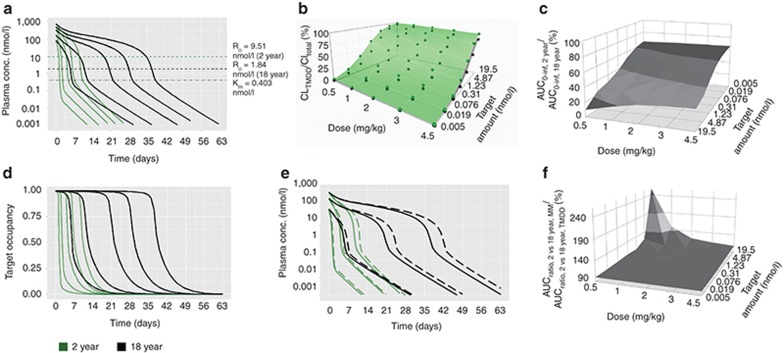
Same target amount, BW-based i.v. dosing. (**a**) Simulated plasma PK profiles for 2-year (green lines) vs. 18-year (black lines) age group (reference group) based on the full TMDD model at doses of 0.5, 1, 2, 3, and 4.5 mg/kg (from left to right. (**b**) CL_TMDD_/CL_total_ for 2-year (green dots connected with the green surface) vs. 18-year (black dots connected with the gray surface) age group. (**c**) AUC_0–infinity_ ratios for 2-year vs. 18-year age group. (**d**) Simulated target occupancy for 2-year (green lines) vs. 18-year (black lines) age group at doses of 0.5, 1, 2, 3, and 4.5 mg/kg (from left to right). (**e**) Comparison of simulated plasma PK profiles by the full TMDD model (solid line) and its Michaelis-Menten approximations (dashed line) in the 2-year (green lines) and 18-year (black lines) age group at doses of 0.5, 2, and 4.5 mg/kg (from left to right). (**f**) Relative change in AUC ratios between 2-year-olds and 18-years olds when comparing MM approximations and full TMDD model. The target amount of 4.87 nmol is equivalent to target concentration (1.74 nmol/l) reported in Luu *et al.*^31^ Sensitivity analysis of the target amount (19.5, 4.87, 1.23, 0.31, 0.076, 0.019, and 0.005 nmol) was performed based on the choice of *R*_0_, which was changed in fourfold increments (6.96, 1.74, 0.44, 0.11, 0.027, 0.0068, and 0.0017 nmol/l).

When total target amount (i.e., higher target concentration of 9.51 nmol/l in pediatrics compared to 1.84 nmol/l in adults by virtue of smaller body volume) was the same between all age groups, BW-based dosing resulted in a rapid decline in systemic mAb concentrations in 2-year-olds compared to 18-year-olds (**Figure 3a**). Although at higher doses, the mAb concentrations were above the *R*_0_ for the respective age-groups (**Figure 3a**) and were able to saturate the target (**Figure 3d**), the duration of the target saturation was comparatively short-lived in the 2-year age group (days) when compared to the 18-year age group (weeks). CL_TMDD_ accounted for 95% and 87% of CL_TOT_ in 2- and 18-year-olds at the lowest dose, while it accounted for 76% and 52% of CL_TOT_ at the highest dose (**Figure 3b**). The sharp decline in CL_TMDD_ contribution to CL_TOT_ in adults suggested greater target saturation at the highest dose and resulting in AUC ratio between pediatrics and adults to be between 25–40% for all doses (**Figure 3c**). Low systemic exposure in pediatrics resulted in shorter duration of target occupancy at all doses (**Figure 3d**), which is well represented by the predicted plasma PK for both age groups. Target occupancy, however, was above 90% on day 14 in 2- and 18-year-olds at low target amount (0.15 nmol) and at lowest dose (0.5 mg/kg). 2- and 18-year-olds mAb PK profiles were comparable between MM approximations and full TMDD model (**Figure 3e**). MM- or Full TMDD-based AUC ratios between 2- and 18-year-olds were comparable except at low dose and high target amount, where the ratio was ~2.5× higher (**Figure 3f**).

### Same target amount: fixed dosing

**Figure 4:**
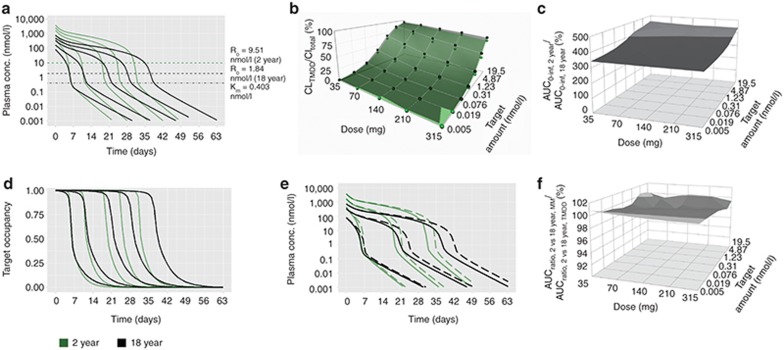
Same target amount, fixed i.v. dosing. (**a**) Simulated plasma PK profiles for 2-year (green lines) vs. 18-year (black lines) age group (reference group) based on the full TMDD model at doses of 35, 70, 140, 210, and 315 mg (from left to right). (**b**) CL_TMDD_/CL_total_ for 2-year (green dots connected with the green surface) vs. 18-year (black dots connected with the gray surface) age group. (**c**) AUC_0–infinity_ ratios for 2-year vs. 18-year age group. (**d**) Simulated target occupancy for 2-year (green lines) vs. 18-year (black lines) age group at doses of 35, 70, 140, 210, and 315 mg (from left to right). (**e**) Comparison of simulated plasma PK profiles by the full TMDD model (solid line) and its Michaelis-Menten approximations (dashed line) in the 2-year (green lines) and 18-year (black lines) age group at doses of 35, 140, and 315 mg (from left to right). (**f**) Relative change in AUC ratios between 2-year-olds and 18-years olds when comparing MM approximations and full TMDD model. The target amount of 4.87 nmol is equivalent to target concentration (1.74 nmol/l) reported in Luu *et al.*^31^ Sensitivity analysis of the target amount (19.5, 4.87, 1.23, 0.31, 0.076, 0.019, and 0.005 nmol) was performed based on the choice of *R*_0_, which was changed in fourfold increments (6.96, 1.74, 0.44, 0.11, 0.027, 0.0068, and 0.0017 nmol/l).

Fixed dosing resulted in higher *C*_max_ values in pediatrics due to their smaller volume of distribution (**Figure 4a**). A rapid decline in mAb plasma concentrations in pediatrics was observed compared to 18-year-olds due to higher target concentration in pediatrics. Contribution of CL_TMDD_ to CL_TOT_ was similar between adults and pediatrics across all doses (**Figure 4b**). Although AUC in pediatrics was consistently higher than adults across all doses and target amounts (**Figure 4c**), the duration of target occupancy was similar between the 2-year and 18-year-olds, especially at lower doses (**Figure 4d**). Plasma mAb PK between MM approximations full TMDD model were reasonably similar for both age groups (**Figure 4e**). AUC ratios obtained from MM or Full TMDD approaches for 2- and 18-year-olds were close to 100% across all doses and target amounts (**Figure 4f**).

## Discussion

Allometric scaling has been successfully employed for small molecules to scale PK parameters from preclinical animal studies to humans using a BW-based power function with a fixed exponent of 0.75 for systemic metabolic clearance and an exponent of 1 for volume of distribution,^21,22^ although the use of a single fixed exponent value for scaling CL has been questioned.^23,24^ For mAbs, different exponents for scaling from non-human primate(s) to humans have been estimated (i.e., 0.75–0.96 for clearance and 1.0–1.12 for volume of distribution^10,25,26,27,28^). Data from non-human primate(s), for mAbs, whose clearance is similar to endogenous IgG (3–5 ml day^−1^ kg^−1^, i.e., linear),^25,26^ is frequently used to predict human PK parameters in the presence of anatomical, physiological, and biochemical similarity and in the absence of nonlinearity.^8^ However, the situation becomes more complex once TMDD significantly contributes to the overall mAb clearance. In these cases, allometric scaling approaches may fail to accurately predict human drug exposure^29^ due to different target affinities,^8^ different target expression levels and turnover rates as they may not be proportional to body size.^30^ The same rationale applies for scaling within species, for example, from adults to children.^15^

The objective of our study was, therefore, to evaluate the impact of differences in target expression between adults and children on pediatric dosing via simulations for mAbs that exhibit nonlinear kinetics. To this end, we first identified a published TMDD model for an anti-ALK1 receptor IgG2 antibody,^31^ which was then used for a model-based comparison of BW-based and fixed dosing regimens for either identical target concentrations or amounts in adults and children, respectively.

There are several key factors that drive clearance and, thus, the exposure of mAbs that exhibit nonlinear kinetics. These factors can be further divided into parameters that determine the linear (CL_Linear_) and those that determine the non-linear component (CL_nonlinear_) of the overall clearance (CL_TOT_; cf. Eq. 5). CL_TOT_ becomes linear when mAb concentrations are either high enough to saturate all targets (DR~*R*_0_) or when mAb concentrations are significantly lower than *K*_m_.^11,14^ Nonlinearity, on the other hand, is most prevalent at the intermediate concentration range when targets are only partially saturated. In order to sufficiently characterize CL_nonlinear_, several factors have to be considered (cf. Eq. 5). While *k*_on_, *k*_off_, and *V*_c_ can be measured quite effectively in either *in vitro* or *in vivo* experiments, target expression as well as respective turnover and internalization rates are typically not readily accessible in clinical settings. This poses a particular challenge as these parameters drive nonlinearity. Given that *k*_int_ characterizes the internalization process on the cellular level, we assumed for our analysis that there are no differences in *k*_int_ between adults and children provided that we did not find any evidence for it to be the case in the literature. Thus, the concentration/amount of target expressed on a whole body level will be more informative of potential differences between adults and children.

Our results indicate that mAbs that exhibit TMDD, when the target concentration is the same between adults and pediatrics, the AUC_0–infinity, children_/AUC_0–infinity, adult_ approaches unity with increasing target concentration, decreasing dose and increasing age (weight) under BW-based i.v. dosing (**Figure 1c**). The underlying mechanism for this finding is that the CL_TMDD_ in this scenario is directly proportional to BW for each age group. When CL_TMDD_ accounts for a major portion of CL_TOT_ (i.e., at low doses, **Figure 1b**), the weight-disproportional CL_Linear_ has less impact on drug exposure. If CL_Linear_ is directly proportional to BW (*b* = 1), little differences in drug exposure are to be expected when dosing on a per kg basis, even in the presence of TMDD. However, fixed dosing resulted in increased systemic mAb exposure (**Figure 2c**) and prolonged target occupancy in pediatrics (**Figure 2d**), with decreasing age, decreasing dose and increasing target concentration. Observed differences in target occupancy between different age groups are associated with higher *C*_max_ in pediatrics due to smaller volume of distribution, even though the target concentration is the same.

If, however, the target amount is the same in adults and children, the AUC_0–infinity, children_/AUC_0–infinity, adult_ approaches unity with decreasing target concentration, increasing dose and increasing age (weight) under BW-based i.v. dosing (**Figure 3c**). This also holds true if target amounts are not the same, but overall target concentrations are higher in children than in adults. Fixed dosing on the other hand resulted in increased systemic mAb exposure in children with decreasing age, decreasing dose and increasing target amount (**Figure 4c**). It should be noted, though, that despite different mAb PK, target occupancy is quite similar between 2-year-olds and 18-year-olds especially at lower doses, which suggests that in isolation, plasma PK is not reflective of the underlying dose-concentration-response relationship. Note that tiered fixed dosing^15^ (i.e., a fixed dose for patients in a specified narrow BW range) may be more appropriate rather than using a single dose for all age groups. It should further be noted that in all four main scenarios, AUC_0–infinity, 12 year_/AUC_0–infinity, adult_ approaches unity with either weight-based or fixed dosing, which suggests that mAbs exhibiting TMDD can be dosed in older children (e.g., 6–17 year) similarly to those exhibiting linear PK.^15^

Using either the full TMDD model or its MM approximation, our results (panels e and f in **Figures 1**–**4**) suggest that if mAbs with TMDD are dosed such that concentrations remain significantly higher than the target concentration and *K*_m_ (i.e., in the linear CL range), BW-based dosing may be sufficient to enable comparable drug exposure between adults and pediatrics. However, this dosing approach may still result in under-exposure in children with low weight as was shown for some mAbs (i.e., infliximab)^15^ with linear PK when using an exponent for CL of less than 1. In addition, simulation-based evidence suggests that the TMDD model and its MM approximation^32^ provide similar results for both BW-based or fixed i.v. dosing and, thus, serve as an adequate model to guide dosing. However, the MM model may not fully capture the TMDD properties if the range of drug concentrations is comparable to, or smaller than, the target concentration nor when *R*_TOT_ is not constant.^32,33^ This is particularly important when the drug concentration in different age groups may differ from the respective target concentrations, in which case the MM approximation may predict different AUC_0–infinity, pediatrics_/AUC_0–infinity, adult_ compared to those obtained from the TMDD model.

For mAbs that bind to membrane bound targets, differences in systemic drug exposure under different dosing approaches, based on our simulations, should be related to both CL_Linear_ (*k*_el_) and CL_TMDD_. For example, for a mAb targeting the Type 1 Insulin-like Growth Factor Receptor showing non-linear PK after 3–16 mg/kg dosing, 32% lower drug exposures were observed in the youngest patients (2–6 year) compared to the 12–17 year olds when dose was normalized to body weight (9 mg/kg).^34^ Whether the observed under-exposure in children with low weight for this mAb is mainly related to the mg/kg dosing, which was described as the main reason for the under-exposure of infliximab and tocilizumab in low-weight children,^15^ requires additional insight on the IGFA target expression and its associated clearance in pediatrics.

Target density is not always dependent on body size but more often on disease type, disease severity and receptor/cell turnover rates. For example, tumor size rather than total body size is more reflective of the total amount of targets that are involved in TMDD, which differs from the scenario when a target is expressed on the vascular endothelial cell (i.e., those surrounding an entire organ). Here the target concentration (when normalized to plasma volume) may be comparable between adults and children because of BW-proportional central volume and BW-proportional organ volume. This applies to the mAb used for our studies that target human ALK1 receptor, a cell surface receptor preferentially expressed on endothelial cells as well as various solid human tumors.^31^ For locally expressed membrane bound targets in the extravascular space, the target concentration, when normalized by the volume of the tumor, may be comparable between adult and children if the tumor biology and the tumor volume are the same. However, when normalized to the total organ volume, or to the central plasma volume, the target concentration should be higher in children when the target amount is the same due to smaller total organ or plasma volume in children. Additional modeling and simulation is needed to address how local targets impact systemic and target site drug exposure. Further research is needed to quantify target concentrations and compare adult and pediatric systemic and ideally target site drug exposures at a wide range of dose levels.

Therefore, the comparison of efficacious drug concentrations in adult in relation to target baseline concentration (*R*_0_) and target affinities (*K*_m_) in adult, together with quantification of *R*_0_ and *K*_m_ in the pediatric population is important for assessing dosing strategies in pediatrics. Sufficient knowledge about the target's localization (i.e., plasma or interstitial fluid (ISF)) is an additional factor that needs to be considered when attempting to optimize target site concentrations due to the significantly reduced drug concentration in the ISF estimated for a number of mAbs.^35^ Whether *R*_0_ should be scaled depends on what is known experimentally about the specific target in question, although there are challenges, such as difficulty in quantitatively estimating total number of cells carrying a particular receptor and unrealistic values of receptor abundance by multiplying the cell-surface density to the total number of cells,^31^ which may reflect limited mAb distribution in the receptor-expressing tissues.^36,37^ When experimental measurements are not available, using the same value of plasma volume normalized *R*_0_ may be applicable to cell surface targets that are widely expressed throughout the body or circulating soluble targets in the central compartment for adults and pediatrics.

Despite the many unknowns (e.g., targets, local drug concentration in children), one practical consideration for mAb dosing is to evaluate the therapeutic doses and resulted plasma concentrations in adults. Antibodies often have effective blood concentrations above 10 μg/ml (i.e., >10 nmol/l), while their target affinities are more often around 1 nmol/l.^38^ The majority of marketed antibody-based therapeutic proteins have a ratio of plasma concentration at clinical dose/K_d_ (equilibrium dissociation constant between the antibody and its antigen) greater than tenfold, with many over 100- to 5,000-fold,^10^ the impact of which has been demonstrated for panitumumab and cetuximab, where their therapeutic concentrations saturate their target.^39,40^ It should further be noted that the concentration of target relative to that of the drug at the target site will also impact target occupancy. Respective PK profiles are consistent with our simulation that when targets are saturated, the PK behavior of mAbs should no longer be affected by the target levels. In a study, where 27 children and 19 adolescents received a median of 7.1 and 6.0 weeks of cetuximab therapy (250 mg/m^2^ weekly), respectively, cetuximab demonstrated dose-dependent non-linear clearance in both children and adolescents,^41^ similar to those in adults.^42^ This may further suggest the utility of BW-based dose when the efficacious concentrations completely saturate the targets.

In summary, our analysis indicates that mechanism-based modeling and simulation approaches^4,6,15,43,44,45^ are valuable tools for selecting mAb dosing regimens and guiding Phase 1 and Phase 2 dose-finding trials in pediatrics, particularly when therapeutic doses in adults lead to non-target saturating drug concentrations. It is further important to interpret PK information in a PK/PD context when attempting to scale adult doses to children as, in isolation, concentrations in blood or plasma may or may not be reflective of the pharmacodynamic response (i.e., target occupancy). Michaelis-Menten approximation of the TMDD model can be effectively utilized under various conditions to characterize the effect of TMDD in pediatrics, such as at target saturating doses. Additionally, not yet evaluated in this study, the impact of age on PK/PD relationships, absorption/disposition characteristics after dosing (i.e., subcutaneous and intramuscular), FcRn maturation and immunogenicity of antibody-based therapeutics, as well as age-related disease differences in both adult and children are relevant for determining the appropriate doses in pediatrics and thus need to be further studied in the future.

## Methods

**Figure 5:**
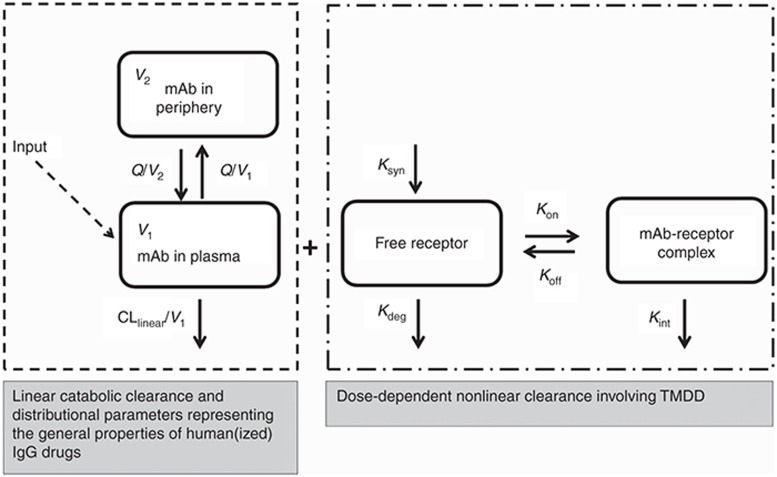
General pharmacokinetic model of target-mediated drug disposition. Adopted from Mager and Jusko.^11^ Symbols are defined in the text.

The conceptual TMDD model proposed by Mager and Jusko^11^ (**Figure 5**) describes the formation of the mAb-receptor complex which is driven by the availability of both free mAb and free target. Degradation of both; membrane-bound and soluble target is characterized by *k*_int_. Mathematically, this scheme of reactions translates into the following set of differential Eqs. 1–4 and the relationship between parameters is shown in Eq. 5:^11^

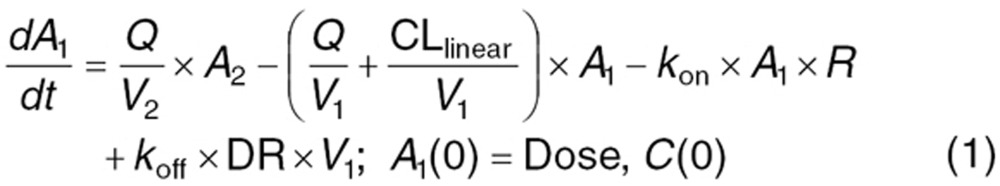


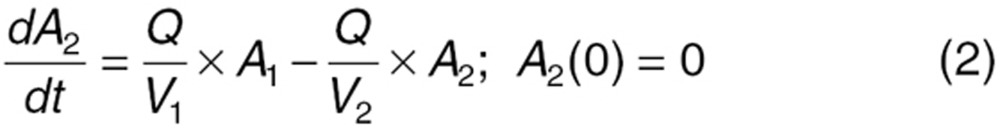


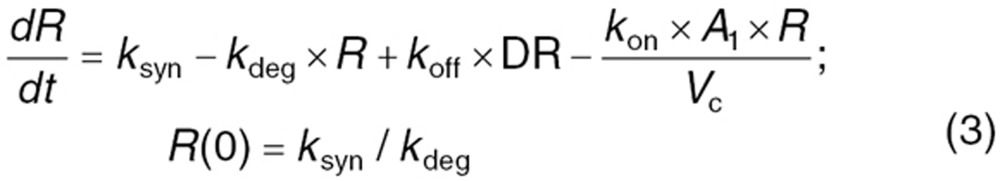


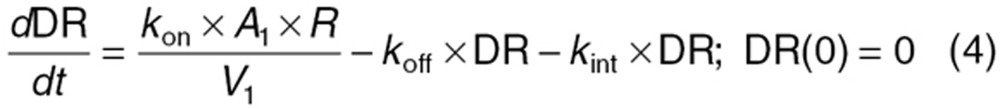


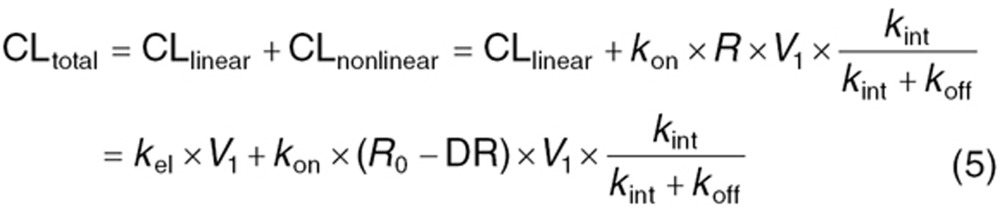


where *A*_1_ and *A*_2_, free mAb amount in the serum and peripheral tissue, respectively; *V*_1_ and *V*_2_, volumes of distribution in the central (plasma) and peripheral compartments respectively; *R*, unbound target concentration; DR, drug-target complex concentration; *Q*, distributional clearance between central (plasma) and peripheral compartments; CL_linear_, linear catabolic clearance; *k*_el_, first-order elimination rate constant of mAb; *k*_syn_, zero-order target synthesis rate constant; *k*_deg_, first-order target degradation rate constant; *k*_int_, first-order internalization rate constant of the drug-target complex; *k*_on_, target binding association rate constant; and *k*_off_, target binding disassociation rate constant.

The endogenous free target concentration at steady-state (*R*_0_) is defined as *k*_syn_/*k*_deg_, total drug concentration as *C*_TOT_, free drug concentration (*C*) + DR; as *R*_TOT_ = *R* + DR, and target occupancy as DR/*R*_TOT_ (see **Supplementary Data** online).

### Model based comparison of different dosing rationales

**Figure 6:**
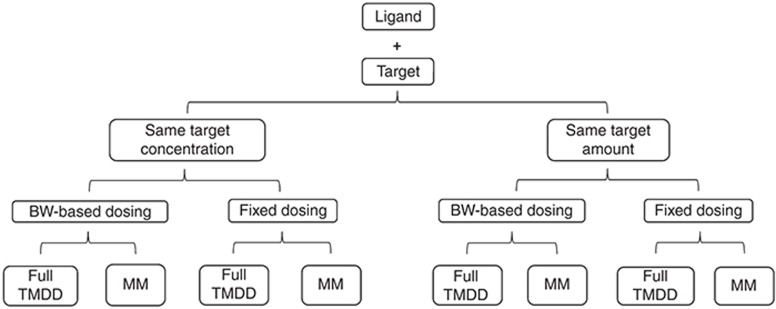
Hierarchical model simulation scenarios. BW, body weight; MM, Michaelis-Menten approximation of TMDD; TMDD, target-mediated drug disposition.

A previously qualified TMDD model and its parameters for an anti-ALK1 receptor mAb^31^ in adults was adopted in our study. CL_Linear_ and *V*_d_ were allometrically scaled based on BW using fixed exponents of 0.75 and 1, respectively, to account for age-dependent differences between adults and children (cf. **Figure 5**). A set of hierarchical simulations (**Figure 6**) was subsequently performed to compare: (i) same target concentration vs. same target amount in adults and children, (ii) BW-based vs. fixed dosing, and (iii) Full TMDD vs. MM approximation modeling approaches. Physiologically relevant target concentrations of 1.74 nmol/l in adults^31^ were used to evaluate same target concentration between adults and children. Target amounts were calculated from target concentrations using a plasma volume of 2.8 liter in a 70 Kg adult. Sensitivity analysis was performed for target concentrations and amounts to determine the impact of changes in target concentrations (6.96, 1.74, 0.44, 0.11, 0.027, 0.0068, and 0.0017 nmol/l) or amounts (19.5, 4.87, 1.23, 0.31, 0.076, 0.019, and 0.005 nmol) on the PK of free drug and drug-target complex.

Single, intravenous (i.v.) bolus mAb dosing was used in our analysis. 1:1 mAb-target binding was assumed (molar units). Feedback due to changes in target synthesis or degradation was not considered. Drug PK profiles and target occupancy (DR/(R + DR)) were simulated in both adults and children based on published doses of 0.5, 1, 2, 3, or 4.5 mg/kg for BW-based dosing scenario (3.33, 6.67, 13.3, 20, and 30 nmol/kg, respectively, using mAb molecular weight of 150 kD).^31^ Mean BW of 12.8, 21.3, 43.5, and 66.1 kg were selected for ages between “2–2.49”, “6.0–6.49”, “12.0–12.49,” and “18.0–18.49” years, respectively (ref. CDC growth chart). Fixed dosing was evaluated using doses of 35, 70, 140, 210, and 315 mg which correspond to 0.5, 1, 2, 3, and 4.5 mg/kg dosing for a 70 kg subject.

Full TMDD-based model predictions were subsequently compared to the corresponding MM approximations as described by Gibiansky *et al.*^32^ *V*_max_ and *K*_m_ were computed as 7.6038 nmol/l/day and 0.403 nmol/l, respectively. *V*_max_ values for each age group were calculated accordingly to reflect either the same target concentration or same target amount. AUC_0–infinity_ values between the two modeling approaches were also compared for every age group.

To evaluate the impact of the different dosing strategies on PD, simulated PK profiles for free mAb concentrations were placed into a PK/PD context by comparing calculated target occupancies at the extremes of the selected age group (18 years and 2 years). Non-compartmental analysis was performed in Phoenix (v 6.3.0.395, Pharsight) to compare systemic mAb exposure in adults and children. Total clearance (CL_TOT_ = Dose/AUC_0–infinity_), CL_Linear_ and target-mediated clearance (CL_TMDD_ = CL_TOT_ − CL_Linear_) of mAb in adults and pediatrics at different dosing groups were also compared. The CL_TMDD_/CL_TOT_ ratios were calculated for both age groups.

### Software for modeling and simulation

Simulations were performed in NONMEM (v 7.2.0, ICON, Dublin, Ireland) with PsN (v 3.6.2) and Pirana (v 2.8.0) and data visualized in R (v 3.0.1, ggplot2), GraphPad Prism (v 5.0, GraphPad Software), Microsoft Excel 2007 (Microsoft Corporation, Seattle, WA), and XLSTAT 2013 (Addinsoft SARL).

## Conflict of Interest

The authors declared no conflict of interest.

## Author Contributions

S.Z., P.G., M.A.A., M.A.G., L.J.L., and S.S. wrote the manuscript. S.Z., P.G., M.A.A., and S.S. designed the research. S.Z., P.G., and S.S. performed the research. S.Z. analyzed the data.

## Study Highlights


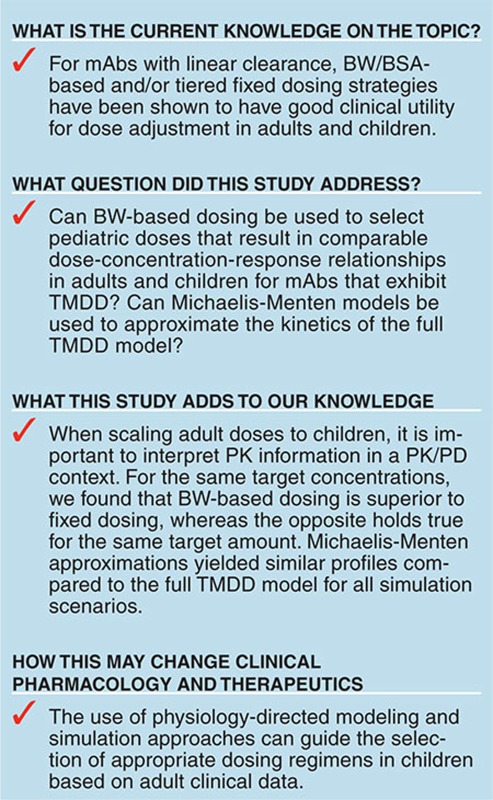


## Figures and Tables

**Table 1:**
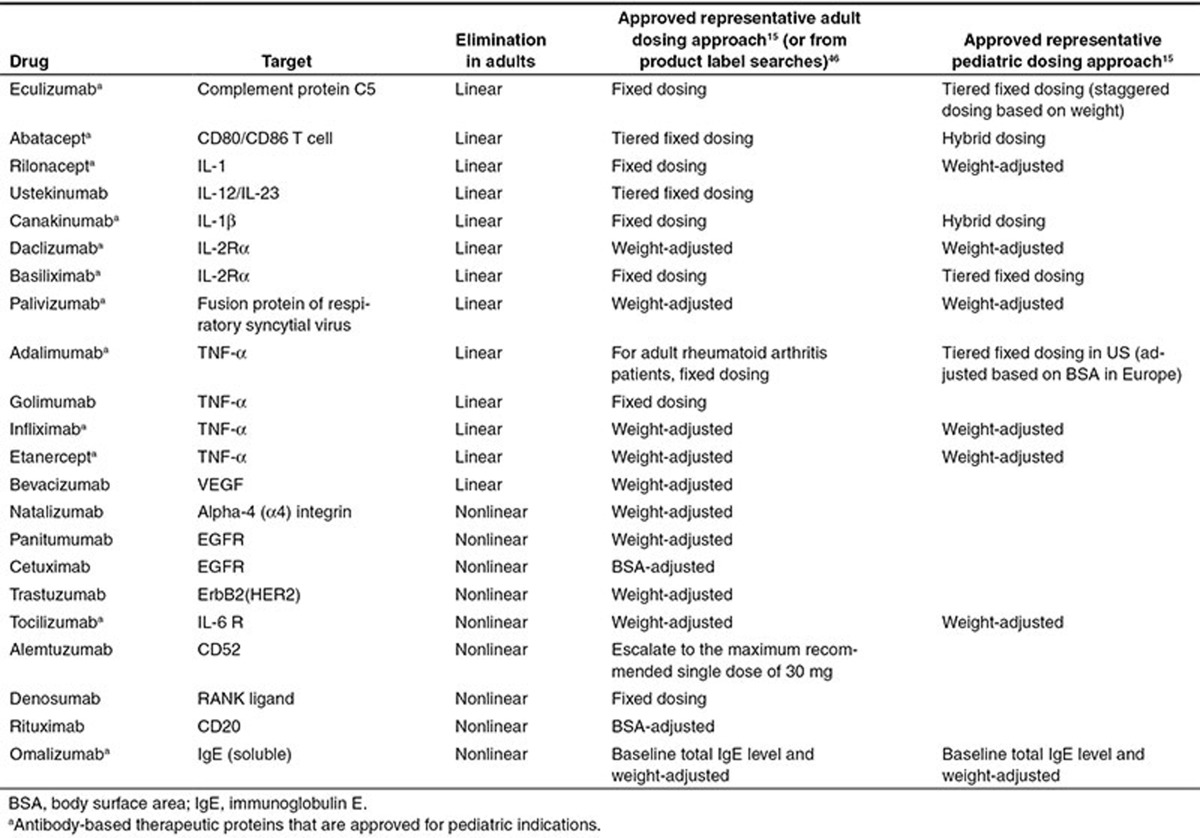
Overview of approved antibody-based therapeutic proteins for application in adults and children
