# Supplementary material for: Model-Based Assessment of Dosing Strategies in Children for Monoclonal Antibodies Exhibiting Target-Mediated Drug Disposition
Source: CPT Pharmacometrics Syst Pharmacol. 2014 Oct 1;3(10):e138–. doi: 10.1038/psp.2014.38 (PMC4474168; doi:10.1038/psp.2014.38)
Supplement: Supplementary Data [file psp201438x1.pdf]

The full TMDD model for simulation (same target concentration case)

```
$PROBLEM     TMDD FULL MODEL IV  DOSING
$INPUT      C ID TIME AMT DV EVID MDV CMT TYPE WT AGE
$DATA  FULLTMDDSINGLEIVDOSE.CSV  IGNORE=C
$SUBROUTINES ADVAN13 TOL=9

;;;;;;;;;;;;;;;;;;;;;;;;;;;;;;;;;;;;;;;;;;;;;;;;;;;;;;;;;;;;;;;;;;;;;;;;;;;;;;;;;;;;;;;;;;;;;;;;;;;;;;;;;;;;;;;;;;;;;;;;;;;;;;
$MODEL
COMP(COMP1);  free drug amount in the plasma  (central compartment)
COMP(COMP2);  free drug amount in the peripheral compartment
COMP(COMP3);  free target concentration
COMP(COMP4);  drug-target complex concentration

;;;;;;;;;;;;;;;;;;;;;;;;;;;;;;;;;;;;;;;;;;;;;;;;;;;;;;;;;;;;;;;;;;;;;;;;;;;;;;;;;;;;;;;;;;;;;;;;;;;;;;;;;;;;;;;;;;;;;;;;;;;;;;
$PK
CL = THETA(1)*EXP(ETA(1))*(WT/70)**0.75
V1 = THETA(2)*EXP(ETA(2))*(WT/70)
Q  = THETA(3)*EXP(ETA(3))*(WT/70)**0.75
V2 = THETA(4)*EXP(ETA(4))*(WT/70)
KEL = CL/V1
K12 = Q/V1
K21 = Q/V2
RMAX = THETA(5)*EXP(ETA(5))
KON  = THETA(6)*EXP(ETA(6))
KOFF  = THETA(7)*EXP(ETA(7))
KDEG = THETA(8)*EXP(ETA(8))
KINT = THETA(9)*EXP(ETA(9))
KSYN = RMAX*KDEG

;;;;;;;;;;;;;;;;;;;;;;;;;;;;;;;;;;;;;;;;;;;;;;;;;;;;;;;;;;;;;;;;;;;;;;;;;;;;;;;;;;;;;;;;;;;;;;;;;;;;;;;;;;;;;;;;;;;;;;;;;;;;;;
$DES
DADT(1)= K21*A(2)-(KEL+K12)*A(1)-KON*A(1)*A(3)+KOFF*A(4)*V1
DADT(2)= K12*A(1)-K21*A(2)
DADT(3)= KSYN-KDEG*A(3)+KOFF*A(4)-KON*A(1)*A(3)/V1
DADT(4)= KON*A(1)*A(3)/V1-KOFF*A(4)-KINT*A(4)

;;;;;;;;;;;;;;;;;;;;;;;;;;;;;;;;;;;;;;;;;;;;;;;;;;;;;;;;;;;;;;;;;;;;;;;;;;;;;;;;;;;;;;;;;;;;;;;;;;;;;;;;;;;;;;;;;;;;;;;;;;;;;;
$ERROR
IPRED=0
I1=0
I2=0
I3=0

IF(TYPE==1) THEN
I1=1
IPRED=A(1)/V1;
ENDIF

IF(TYPE==3) THEN
I2=1
IPRED=A(3);
ENDIF

IF(TYPE==4) THEN
I3=1
IPRED=A(4);
ENDIF

Y= IPRED*(1+EPS(1)*I1+EPS(2)*I2+EPS(3)*I3)
```

```
TAROCC=A(4)/(A(3)+A(4)); Target Occupancy

;;;;;;;;;;;;;;;;;;;;;;;;;;;;;;;;;;;;;;;;;;;;;;;;;;;;;;;;;;;;;;;;;;;;;;;;;;;;;;;;;;;;;;;;;;;;;;;;;;;;;;;;;;;;;;;;;;;;;;;;;;;;;;;;;;
$THETA
(0.1848 FIX);    1 CL
(2.8 FIX);       2 V1
(0.6384 FIX);    3 Q
(3.011 FIX);     4 V2
(1.74 FIX);      5 RMAX
(75.17 FIX);     6 Kon
(25.92 FIX);     7 Koff
(5.36 FIX);      8 KDEG
(4.37 FIX);      9 KINT

;;;;;;;;;;;;;;;;;;;;;;;;;;;;;;;;;;;;;;;;;;;;;;;;;;;;;;;;;;;;;;;;;;;;;;;;;;;;;;;;;;;;;;;;;;;;;;;;;;;;;;;;;;;;;;;;;;;;;;;;;;;;;;;;;;
$OMEGA
0 FIX ;          1 CL
0 FIX;           2 V1
0 FIX;           3 Q
0 FIX;           4 V2
0 FIX;           5 RMAX
0 FIX;           6 Kon
0 FIX;           7 Koff
0 FIX;           8 KDEG
0 FIX;           9 KINT

;;;;;;;;;;;;;;;;;;;;;;;;;;;;;;;;;;;;;;;;;;;;;;;;;;;;;;;;;;;;;;;;;;;;;;;;;;;;;;;;;;;;;;;;;;;;;;;;;;;;;;;;;;;;;;;;;;;;;;;;;;;;;;;;;;
$SIGMA
0 FIX
0 FIX
0 FIX


$SIMULATION (2674474) ONLYSIMULATION SUBPROBLEMS=1

;;;;;;;;;;;;;;;;;;;;;;;;;;;;;;;;;;;;;;;;;;;;;;;;;;;;;;;;;;;;;;;;;;;;;;;;;;;;;;;;;;;;;;;;;;;;;;;;;;;;;;;;;;;;;;;;;;;;;;;;;;;;;;;;;;
$TABLE    C ID TIME AMT IPRED DV TAROCC EVID MDV CMT TYPE WT AGE
NOPRINT    FILE=SDTABFULLTMDDSINGLEIVDOSE001
```

Michaelis-Menten approximation of the full TMDD model for simulation (same target concentration case)

```
$PROBLEM     MM MODEL IV DOSING
$INPUT      C ID TIME AMT DV EVID MDV CMT TYPE WT AGE
$DATA  MMSINGLEIVDOSE.CSV  IGNORE=C
$SUBROUTINES ADVAN13 TOL=9

;;;;;;;;;;;;;;;;;;;;;;;;;;;;;;;;;;;;;;;;;;;;;;;;;;;;;;;;;;;;;;;;;;;;;;;;;;;;;;;;;;;;;;;;;;;;;;;;;;;;;;;;;;;;;;;;;;;;;;;;;;;;;;;;;;
$MODEL
COMP(COMP1);  free drug amount in the plasma  (central compartment)
COMP(COMP2);  free drug amount in the peripheral compartment

;;;;;;;;;;;;;;;;;;;;;;;;;;;;;;;;;;;;;;;;;;;;;;;;;;;;;;;;;;;;;;;;;;;;;;;;;;;;;;;;;;;;;;;;;;;;;;;;;;;;;;;;;;;;;;;;;;;;;;;;;;;;;;;;;;
$PK
CL = THETA(1)*EXP(ETA(1))*(WT/70)**0.75
V1 = THETA(2)*EXP(ETA(2))*(WT/70)
Q  = THETA(3)*EXP(ETA(3))*(WT/70)**0.75
V2 = THETA(4)*EXP(ETA(4))*(WT/70)
KEL = CL/V1
K12 = Q/V1
K21 = Q/V2
RMAX = THETA(5)*EXP(ETA(5))
KON  = THETA(6)*EXP(ETA(6))
KOFF  = THETA(7)*EXP(ETA(7))
KDEG = THETA(8)*EXP(ETA(8))
KINT = THETA(9)*EXP(ETA(9))
KSYN = RMAX*KDEG

VMAX = RMAX*KINT
KM = (KINT+KOFF)/KON

S1=V1

;;;;;;;;;;;;;;;;;;;;;;;;;;;;;;;;;;;;;;;;;;;;;;;;;;;;;;;;;;;;;;;;;;;;;;;;;;;;;;;;;;;;;;;;;;;;;;;;;;;;;;;;;;;;;;;;;;;;;;;;;;;;;;;;;;
$DES
DADT(1)= K21*A(2)-(KEL+K12)*A(1)-VMAX*A(1)/(KM+A(1)/S1)
DADT(2)= K12*A(1)-K21*A(2)

;;;;;;;;;;;;;;;;;;;;;;;;;;;;;;;;;;;;;;;;;;;;;;;;;;;;;;;;;;;;;;;;;;;;;;;;;;;;;;;;;;;;;;;;;;;;;;;;;;;;;;;;;;;;;;;;;;;;;;;;;;;;;;;;;;
$ERROR
IPRED=A(1)/V1
Y= IPRED*(1+EPS(1))
IRES=IPRED-DV

;;;;;;;;;;;;;;;;;;;;;;;;;;;;;;;;;;;;;;;;;;;;;;;;;;;;;;;;;;;;;;;;;;;;;;;;;;;;;;;;;;;;;;;;;;;;;;;;;;;;;;;;;;;;;;;;;;;;;;;;;;;;;;;;;;
$THETA
(0.1848 FIX);    1 CL
(2.8 FIX);       2 V1
(0.6384 FIX);    3 Q
(3.011 FIX);     4 V2
(1.74 FIX);      5 RMAX
(75.17 FIX);     6 Kon
(25.92 FIX);     7 Koff
(5.36 FIX);      8 KDEG
(4.37 FIX);      9 KINT

;;;;;;;;;;;;;;;;;;;;;;;;;;;;;;;;;;;;;;;;;;;;;;;;;;;;;;;;;;;;;;;;;;;;;;;;;;;;;;;;;;;;;;;;;;;;;;;;;;;;;;;;;;;;;;;;;;;;;;;;;;;;;;;;;;
$OMEGA
0 FIX;           1 CL
0 FIX;           2 V1
```

```
0 FIX;          3 Q
0 FIX;          4 V2
0 FIX;          5 RMAX
0 FIX;          6 Kon
0 FIX;          7 Koff
0 FIX;          8 KDEG
0 FIX;          9 KINT

;;;;;;;;;;;;;;;;;;;;;;;;;;;;;;;;;;;;;;;;;;;;;;;;;;;;;;;;;;;;;;;;;;;;;;;;;;;;;;;;;;;;;;;;;;;;;;;;;;;;;;;;;;;;;;;;;;;;;;;;;;;;;;;;;;;;;;;
$SIGMA
0 FIX

$SIMULATION (2674474) ONLYSIMULATION SUBPROBLEMS=1

;;;;;;;;;;;;;;;;;;;;;;;;;;;;;;;;;;;;;;;;;;;;;;;;;;;;;;;;;;;;;;;;;;;;;;;;;;;;;;;;;;;;;;;;;;;;;;;;;;;;;;;;;;;;;;;;;;;;;;;;;;;;;;;;;;;;;;;
$TABLE    C ID TIME AMT IPRED IRES DV EVID MDV  CMT TYPE WT AGE
NOPRINT    FILE=SDTABMMSINGLEIVDOSE001
```
